# Cyclic di-GMP Regulation of Antibiotic-Induced Biofilm Formation: A Systematic Review

**DOI:** 10.3390/antibiotics15101023

**Published:** 2026-10-15

**Authors:** Md. Ekramul Karim, Taryn Eubank, Khurshida Begum, M. Jahangir Alam, Julian G. Hurdle, Kevin W. Garey

**Affiliations:** 1 Department of Pharmacy Practice and Translational Research, University of Houston, Houston, TX 77204, USA; mkarim2@uh.edu (M.E.K.); taeubank@central.uh.edu (T.E.); kbegum@central.uh.edu (K.B.); mjalam@central.uh.edu (M.J.A.); 2 Department of Biology & Biochemistry, University of Houston, Houston, TX 77204, USA; 3 Center for Infectious and Inflammatory Diseases, Institute of Biosciences and Technology, Department of Translational Medical Sciences, Texas A&M Health Science Center, Houston, TX 77030, USA; jhurdle@tamu.edu

**Keywords:** biofilm, sub-MIC, c-di-GMP, diguanylate cyclase, phosphodiesterase, biofilm infection, *Clostridioides difficile* infection

## Abstract

**Background/Objectives**: Biofilm formation is a major bacterial survival strategy in response to antibiotic stress. The bacterial second messenger cyclic di-GMP (c-di-GMP) is a central regulator of biofilm development. Sub-minimum inhibitory concentrations (sub-MICs) of antibiotics act as biological active environmental cues that reprogram bacterial physiology and promote biofilm development. This systematic review synthesizes the current literature on sub-MIC antibiotic-induced biofilm formation across different pathogenic bacterial genera and critically evaluates the role of c-di-GMP. **Methods**: A systematic literature search was conducted in PubMed and Scopus for articles published from inception to 18 December 2025 using terms including “sub-MIC antibiotic” AND (“biofilm” OR “antibiotic-induced biofilm”) and a second search using “sub-MIC antibiotic” AND “biofilm” AND “c-di-GMP”. Reviews, commentaries, opinions, meta-analyses, systematic reviews, and non-English-language articles were excluded. **Results**: Collectively, 28 identified studies demonstrate that sub-MIC antibiotics modulate biofilm formation through diverse mechanisms that intersect with c-di-GMP activity, including modulation of diguanylate cyclases and phosphodiesterases and through regulatory networks that intersect with quorum sensing and different two-component systems. These responses influence multiple components of biofilm matrix- and biofilm-associated physiology, including exopolysaccharides, extracellular DNA, cell surface proteins, secretion systems, and stress-adaptive multicellular behavior. **Conclusions**: By positioning c-di-GMP as an integrative, but context-dependent, signaling hub linking antibiotic-induced stress, metabolic adaptation, persistence, and biofilm development, this review highlights critical mechanistic gaps and identifies potential therapeutic targets for disrupting biofilms and improving antibiotic efficacy.

## 1. Introduction

Biofilms represent a dominant bacterial lifestyle and are a major contributor to community- and healthcare-associated infections [1]. Biofilms are implicated in more than 80% of microbial infections and over 60% of hospital-associated infections [2,3]. Bacterial biofilms consist of single or multispecies communities embedded within an extracellular matrix composed of polysaccharides, extracellular DNA (eDNA), proteins, and lipids [1]. Within biofilms, bacterial cells exist in highly structured and physiologically heterogeneous microenvironments that provide protection against environmental and host-associated stresses, including antibiotic exposure and immune clearance [4]. Biofilm-associated bacteria have a 10–1000-times reduction in antibiotic susceptibility compared to their planktonic counterparts [5]. Biofilm-associated antibiotic tolerance includes restricted antibiotic penetration in addition to nutrient and oxygen gradients within mature biofilms [6]. These generate physiological and metabolic heterogeneity resulting in subpopulations of slow-growing or metabolically altered cells with enhanced tolerance to antibiotic stress [7]. Thus, biofilm-associated antibiotic tolerance emerges from structural protection along with stress-adaptive physiological and metabolic states.

Antibiotics administered at sub-minimum inhibitory concentrations (sub-MICs) can actively induce biofilm formation across diverse bacterial pathogens [8,9,10,11,12,13,14,15,16,17,18,19,20,21,22,23]. Sub-MIC antibiotic exposure functions as a biologically relevant environmental cue that reshapes bacterial physiology, metabolism, and regulatory behavior [24]. Sub-MIC antibiotics can alter central metabolism, respiratory activity, redox balance, and stress-response pathways, collectively shifting bacterial populations away from rapid proliferation toward stress-adapted and biofilm-associated states [25]. These physiological changes are increasingly linked to activation of global regulatory systems, including quorum sensing, stringent response signaling and second messenger signaling-associated regulatory pathways [26,27]. Nevertheless, the molecular mechanisms connecting sub-MIC antibiotic exposure and biofilm development remain incompletely understood [10,19,21,22,28].

The bacterial second messenger bis-(3′-5′)-cyclic dimeric guanosine monophosphate (c-di-GMP) is a central regulator of biofilm formation. c-di-GMP regulates a broad range of bacterial processes, including motility, surface adaptation, biofilm development, cell cycle progression, and virulence [26]. Intracellular c-di-GMP levels are dynamically controlled by diguanylate cyclases (DGCs) and phosphodiesterases (PDEs), which synthesize and degrade c-di-GMP, respectively [29]. Through binding with diverse effector molecules such as riboswitches, transcription factors, enzymes, and multiprotein complexes, c-di-GMP exerts regulatory control at transcriptional, post-transcriptional, and post-translational levels [29]. Elevated intracellular c-di-GMP levels favor sessile, biofilm-associated states, whereas reduced c-di-GMP levels promote planktonic growth and motility. For example, planktonic *Pseudomonas aeruginosa* cells contain less than 30 pmol/mg c-di-GMP, whereas biofilm-associated cells exhibit concentrations of approximately 75–100 pmol/mg [30].

Many hallmark biofilm-associated traits, including exopolysaccharide (EPS) production, eDNA release, type IV pili and fimbriae expression, surface adhesin regulation, antibiotic tolerance, secondary metabolite production, and biofilm dispersal, are regulated directly or indirectly by c-di-GMP signaling [26]. Regulatory systems activated during antibiotic stress including quorum sensing pathways, two-component systems, and stress-response networks intersect with c-di-GMP-dependent signaling [24]. Consequently, c-di-GMP is increasingly viewed not simply as a biofilm-promoting second messenger, but as an integrative signaling hub that coordinates environmental sensing, physiological adaptation, and multicellular behavior during biofilm development. Understanding how sub-MIC antibiotics interface with c-di-GMP signaling networks to promote biofilm formation is critical for predicting bacterial adaptation during antimicrobial exposure and for developing strategies to prevent or disrupt biofilm-associated infections. This systematic review synthesizes current evidence on sub-MIC antibiotic-induced biofilm formation across diverse bacterial pathogens, with particular emphasis on the role of c-di-GMP signaling in mediating these responses.

## 2. Methods

A systematic literature search was conducted in PubMed and Scopus for articles published up to 18 December 2025 using the following two search strategies: (1) “sub-MIC antibiotic” AND (“biofilm” OR “antibiotic-induced biofilm”) and (2) “sub-MIC antibiotic” AND “biofilm” AND “c-di-GMP”. Reviews, commentaries, opinions, meta-analyses, systematic reviews, and non-English-language articles were excluded. For this review, sub-MIC was defined as any antibiotic concentration below the minimum inhibitory concentration (MIC). Included studies evaluated a broad range of sub-MICs, from approximately 1/70× MIC to 0.75× MIC. Although concentrations ≤ 0.25× MIC are generally considered more conservative because MIC determinations are subject to inherent twofold dilution variability, studies employing higher sub-MICs (e.g., 0.5× or 0.75× MIC) were also included to comprehensively capture the diversity of experimental approaches used to investigate antibiotic-induced biofilm formation. Original research articles were eligible for inclusion if they (1) reported biofilm induction in response to sub-MICs of antibiotics in bacterial pathogens and (2) provided a defined mechanistic framework for biofilm formation. Mechanistic evidence was defined a priori as either direct demonstration of c-di-GMP involvement (e.g., measurement of intracellular c-di-GMP levels, identification of DGCs, PDEs, riboswitches, or c-di-GMP-binding effectors) or indirect evidence through regulatory pathways known to be functionally linked to c-di-GMP signaling, including quorum sensing, stress-response systems, two-component regulatory systems, or biofilm phenotypes previously reported to be c-di-GMP-dependent. Studies reporting only descriptive increases in biofilm biomass following sub-MIC antibiotic exposure without mechanistic insight into biofilm regulation were excluded. Two investigators (MEK and TE) independently screened titles and abstracts for eligibility, followed by full-text review. Discrepancies were resolved by discussion, with input from all investigators when necessary. The review was conducted in accordance with the Preferred Reporting Items for Systematic Reviews and Meta-Analyses (PRISMA) guidelines [31]. This review was not registered, and a formal protocol was not prepared.

## 3. Results

### 3.1. Study Selection and Characteristics

**Figure 1:**
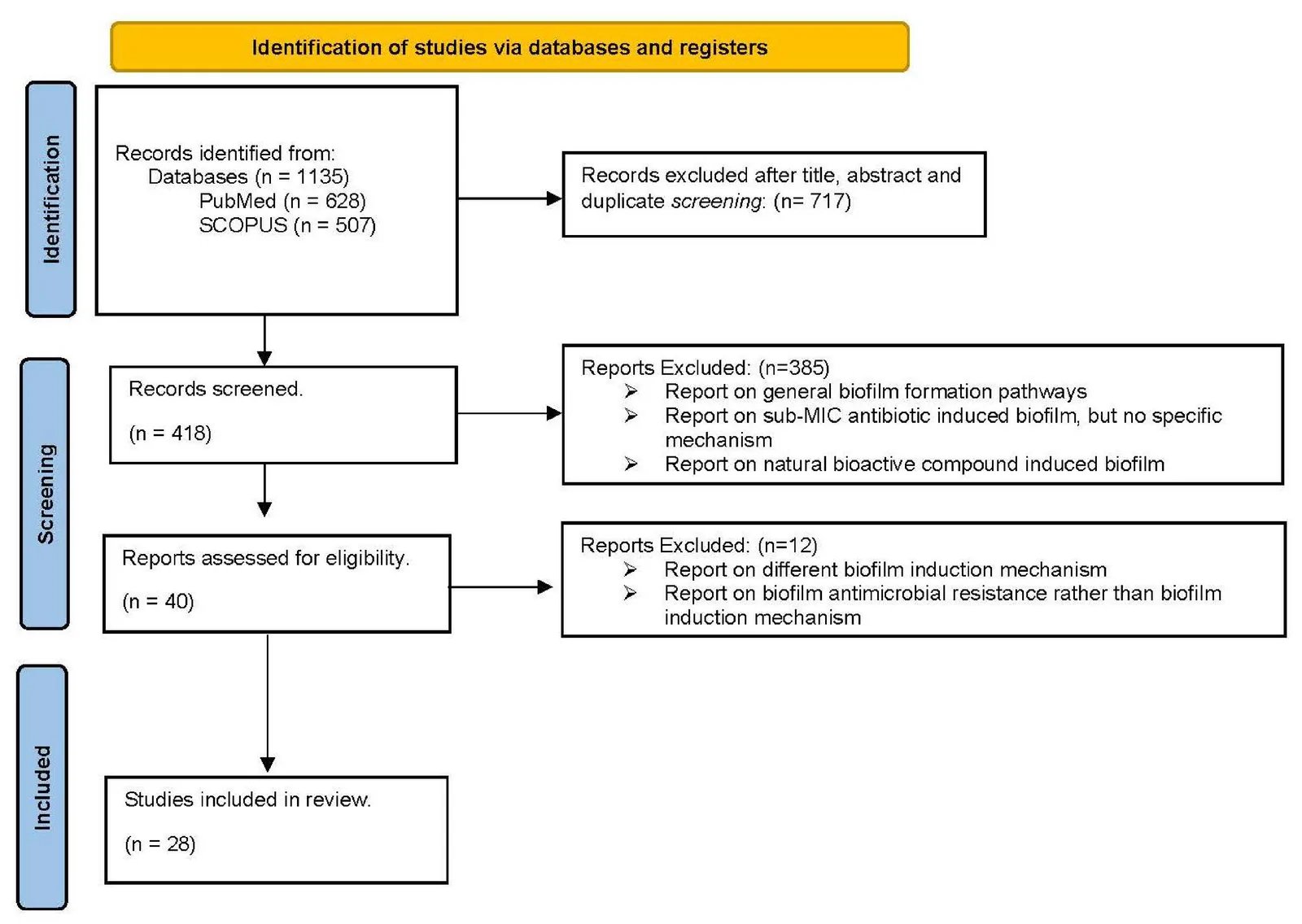
PRISMA flow chart of systematic reviews (http://www.prisma-statement.org/, accessed on 2 January 2025).

The PRISMA flow chart is shown in Figure 1. An initial search of both databases yielded 1135 articles based on predefined search criteria. After removal of duplicates and application of inclusion and exclusion criteria, 28 English-language primary research articles were retained for analysis [8,9,10,11,13,14,15,18,19,21,22,28,32,33,34,35,36,37,38,39,40,41,42,43,44,45,46,47]. All included studies were laboratory-based experimental investigations, predominantly in vitro, employing planktonic and surface-attached biofilm models (e.g., microtiter plate assays, flow cells, chemostat systems). No animal or human clinical interventional studies directly assessing sub-MIC antibiotic-induced biofilm formation were identified. Several studies utilized clinical isolates. The selected studies examined a broad range of bacterial pathogens, including *P. aeruginosa*, *Pseudomonas putida*, *Staphylococcus epidermidis*, *Staphylococcus aureus*, *Enterococcus faecalis*, *Salmonella enterica* serovar Typhimurium, *Escherichia coli*, *Klebsiella pneumoniae*, *Clostridioides difficile*, *Acinetobacter baumannii*, and *Haemophilus influenzae*. Antibiotic classes evaluated included β-lactams, aminoglycosides, tetracyclines, fluoroquinolones, macrolides, glycopeptides, and thiopeptide antibiotics, tested across a wide range of sub-MICs (from ~1/70× MIC to 0.75× MIC).

### 3.2. Global Trends in Sub-MIC Antibiotic-Induced Biofilm Formation

Across the 28 included studies, sub-MIC antibiotic exposure consistently promoted biofilm formation across phylogenetically diverse bacteria by enhancing key biofilm matrix components. These included increased production of EPS (e.g., alginate, cellulose, colanic acid, poly-N-acetylglucosamine (PNAG), and Pel/Psl), elevated eDNA abundance, and increased expression of surface adhesins, including fimbriae or pili involved in initial surface attachment. Gram-negative bacterial results are summarized in Table 1, whereas Gram-positive bacterial results are summarized in Table 2. In Gram-negative bacteria, particularly *P. aeruginosa*, sub-MIC aminoglycosides and β-lactams induced biofilm formation through c-di-GMP-associated regulatory networks, including modulation of diguanylate cyclase/phosphodiesterase activity, altered outer membrane signaling (e.g., OprF-SigX pathways), increased alginate synthesis, and enhanced eDNA release [9,19,28,32,33,34]. Similar c-di-GMP-linked responses were observed in *P. putida*, *E. coli*, and *K. pneumoniae*, where ribosomal stress or antibiotic exposure elevated intracellular c-di-GMP levels, leading to increased polysaccharide production, fimbrial expression and surface adherence [10,11,21,22,35]. In Gram-positive organisms, including *S. epidermidis* and *S. aureus*, sub-MIC antibiotics promoted biofilm formation predominantly through eDNA-dependent mechanisms, increased autolysin activity, and upregulation of surface adhesins [14,15,18,41,42,43,44]. Although these species lack canonical c-di-GMP signaling, several studies identified activation of two-component system (TCS) and global regulatory pathways that functionally converge on biofilm matrix production and stabilization. In contrast, *C. difficile* biofilm exhibited direct c-di-GMP involvement [38,39,40,47]. These findings indicated that Gram-positive bacteria might respond to sub-MIC antibiotic stress through both c-di-GMP-independent and c-di-GMP-linked pathways that converge on stable biofilm formation. Collectively, these data support a unifying model in which sub-MIC antibiotics act as environmental cues that reprogram bacterial lifestyle toward a sessile, biofilm-associated state through c-di-GMP-mediated or c-di-GMP-adjacent regulatory networks.

### 3.3. Species-Specific Mechanisms of Sub-MIC Antibiotic-Induced Biofilm Formation

To contextualize organism-specific responses, the following sections summarize current knowledge of how sub-MIC antibiotics induce biofilm formation across major bacterial pathogens. For each species, the antibiotic classes involved are highlighted, as well as the primary biofilm matrix components affected and whether c-di-GMP regulation has been demonstrated directly (e.g., measured intracellular c-di-GMP levels; identified DGCs/PDEs, riboswitches, or c-di-GMP-binding effectors) or indirectly (e.g., inferred through activation of quorum sensing, stress responses, or biofilm phenotypes known to be c-di-GMP-dependent). This structure facilitates cross-species comparison while emphasizing shared regulatory themes.

### 3.4. Gram-Negative Bacteria: Pseudomonas Species

*Pseudomonas aeruginosa* is a Gram-negative pathogen associated with pneumonia, bacteremia, sepsis, and nosocomial infections. Due to intrinsic antibiotic resistance mediated by the active efflux pumps, mutations to drug targets and acquisition of resistance determinants, *P. aeruginosa* is classified as a WHO high-priority pathogen [48]. Yaeger et al. [19] demonstrated that sub-MICs of cefixime, tobramycin, and thiostrepton induced biofilm formation in *P. aeruginosa* with maximal induction observed at 0.25–0.5× MIC for cefixime and tobramycin. In the same study, genetic screening identified multiple biofilm-deficient mutants, including PA2200, a predicted c-di-GMP phosphodiesterase containing a periplasmic cell-surface signaling (CSS) domain, further supporting a model in which sub-MIC antibiotic exposure perturbs central metabolism and electron transport chain flux, disrupts periplasmic disulfide bonding and cysteine redox homeostasis, and subsequently activates envelope stress signaling pathways converging on c-di-GMP modulation to induce biofilm formation. Hoffman et al. [28] showed that tobramycin 0.3× MIC robustly induced biofilm formation across diverse growth conditions and abiotic surfaces. Transposon mutagenesis identified Arr, a c-di-GMP phosphodiesterase, as a key regulator of aminoglycoside-induced biofilm formation, enabling elevated c-di-GMP in cells. However, Elliot et al. [32] reported that clinical CF isolates lacking *arr* still exhibited tobramycin-induced biofilm formation, indicating the involvement of additional homologous c-di-GMP regulatory pathways. Tahrioui et al. [33] further demonstrated that sub-MIC tobramycin enhances biofilm formation via eDNA release, quorum sensing, and PrrF small regulatory RNAs, linking iron homeostasis and stress signaling to biofilm regulation. Similarly, Kumar et al. [9] demonstrated that sub-MIC gentamicin promoted biofilm formation by increasing eDNA, exoprotein production, reactive oxygen species generation, and cell surface hydrophobicity, suggesting that antibiotic-induced oxidative stress and extracellular matrix remodeling collectively enhance biofilm establishment. Bagge et al. [34] reported that sub-MIC imipenem upregulated alginate biosynthesis genes, correlating with increased alginate production. In *P. putida*, Mu et al. [10] showed that sub-MIC tetracycline activated the RpoS-WspR pathway, elevating intracellular c-di-GMP levels and promoting EPS-dependent biofilm formation. Collectively, these findings indicate that sub-MIC antibiotics function as environmental signals that promote biofilm formation in *Pseudomonas* by activating stress-responsive pathways that converge on c-di-GMP signaling and extracellular matrix production.

### 3.5. Escherichia coli

*E. coli* is a Gram-negative pathogen associated with urinary tract infections, bacteremia, sepsis, and nosocomial infections. Boehm et al. [21] demonstrated that ribosomal stress induced by translation inhibitors (erythromycin, chloramphenicol, tetracycline, and streptomycin) activates c-di-GMP via diguanylate cyclase, YdeH and guanosine pentaphosphate (ppGpp). This in turns increases PNAG production via upregulation of *PgaA* and *PgaD* genes, key PNAG proteins in Gram-negative bacteria. Sailer et al. [11] demonstrated that sub-MIC exposure to the β-lactam antibiotics (carbenicillin, cefotetan, cephaloridine, cephalothin, and ticarcillin) selectively stimulated expression of *cps* operon, essential for colanic acid capsular polysaccharide biosynthesis promoting biofilm maturation. In contrast, *cps* induction was not observed with other β-lactams, including ampicillin, ceftazidime, ceftriaxone, and penicillin. Moreover, antibiotics targeting protein synthesis (chloramphenicol, erythromycin, tetracycline) or DNA synthesis (ciprofloxacin, nalidixic acid, sulfisoxazole) failed to induce *cps* expression, demonstrating that colanic acid-mediated biofilm enhancement represents a non-universal β-lactam-responsive signaling pathway.

### 3.6. Klebsiella pneumoniae

*K. pneumoniae* is a Gram-negative pathogen associated with bacteremia, sepsis, pneumonia, and nosocomial infections. Zhang et al. [22] showed that sub-MIC levofloxacin increased intracellular c-di-GMP levels, resulting in thicker biofilms with elevated EPS and type 3 fimbriae production. This response was associated with increased expression of *yfiN* (c-di-GMP synthesis) and *mrkJ* (c-di-GMP degradation) with preferential upregulation of *yfiN* favoring c-di-GMP accumulation and induction of *mrkH* gene and the *mrkABCDF* fimbrial gene cluster. *MrkH* subsequently activated the mrkA promoter, driving type 3 fimbriae synthesis and biofilm maturation. Guo et al. [35] demonstrated that sub-MIC tetracycline, doxycycline, and minocycline induced biofilm formation in minocycline-resistant clinical isolates, accompanied by increased eDNA release and surface hydrophobicity, as well as significant upregulation of *mrkA*, further reinforcing the central role of type 3 fimbriae and c-di-GMP-associated regulation in antibiotic-induced biofilm development.

### 3.7. Salmonella enterica serovar Typhimurium

*Salmonella enterica* is a Gram-negative pathogen associated with gastroenteritis, bacteremia, typhoid fever, and foodborne infections. Majtán et al. [13] found that cefotaxime 0.5× MIC enhanced biofilm formation and EPS production in multiple *S. enterica* strains. Wang et al. [36] identified the penicillin-binding protein PBP1b (encoded by *mrcB*) as a critical determinant of cefotaxime-induced biofilm formation, linking β-lactam target engagement to biofilm regulation. Collectively, these studies indicate that β-lactam-induced cell wall stress may act as a trigger for biofilm formation in *S. enterica* serovar Typhimurium, although the regulatory networks that translate this signal into enhanced EPS production and biofilm development remain unclear.

### 3.8. Acinetobacter baumannii

*A. baumannii*, a Gram-negative, strictly aerobic, coccobacillus, is a major cause of nosocomial infections, including ventilator-associated pneumonia, bloodstream infections, wound infections, and urinary tract infections [37]. Yamabe et al. [37] showed that sub-MIC cephalosporins enhanced the biomass of pre-established biofilms by increasing eDNA, proteins, lipids, and outer membrane vesicles, indicating matrix remodeling rather than de novo biofilm initiation. These observations indicate that sub-MIC cephalosporins promote persistence of established *A. baumannii* biofilms through antibiotic-induced remodeling of the biofilm matrix, although the signaling networks that link β-lactam exposure to eDNA release and outer membrane vesicle production remain to be elucidated.

### 3.9. Haemophilus influenzae

Nontypeable *H. influenzae* (NTHi) is an opportunistic pathogen associated with otitis-media and respiratory infections [8]. Wu et al. [8] showed that sub-MIC β-lactam antibiotics induced dense biofilms but reduced culturability accompanied by upregulation of glycogen biosynthesis genes and glycogen accumulation. Marti et al. [46] demonstrated that sub-MIC cefuroxime enhanced biofilm formation by disrupting peptidoglycan synthesis leading to bacterial lysis and eDNA release, an effect shared by other β-lactams but not by antibiotics targeting protein synthesis, nucleic acid synthesis, or membrane integrity. Cefuroxime increased eDNA levels nearly tenfold, and DNase treatment abolished biofilm formation in both mutant and wild-type strains. Notably, 13 of 16 clinical isolates exhibited enhanced biofilm formation in response to sub-MIC cefuroxime, indicating that this response is prevalent among NTHi strains.

### 3.10. Gram-Positive Bacteria: Clostridioides difficile

*C. difficile*, a Gram-positive, spore-forming anaerobe, and CDC-designated urgent threat level pathogen, is a leading cause of healthcare-associated infections [49,50]. Dapa et al. [38] showed that vancomycin did not induce biofilm formation after 24h but significantly enhanced biofilm biomass after 72h at 0.5 times the MIC (0.5 μg/mL) and to a lesser extent at 0.25 times the MIC (0.25 μg/mL). Higher concentrations were inhibitory. Vuotto et al. [39] demonstrated that sub-MIC metronidazole enhanced biofilm formation in both susceptible and reduced-susceptible strains. Dawson et al. [40] identified c-di-GMP as a key regulator of early biofilm development, acting through c-di-GMP-dependent surface proteins (CD2831, CD3246) and additional matrix-associated proteins (CD3392, CD0183) with biofilm biomass positively correlating with eDNA abundance across multiple ribotypes (RT012, RT023, RT078, RT027, and RT017). Yang et al. [47] observed elevated intracellular c-di-GMP levels at sub-MIC vancomycin concentrations (0.25× and 0.0625× MIC) in symbiotic biofilms formed between *C. difficile* and *Bacteroides thetaiotaomicron*. Despite these advances, the molecular integration of antibiotic stress, c-di-GMP signaling, and matrix assembly in *C. difficile* biofilms remains incompletely defined.

### 3.11. Staphylococcus epidermidis

*S. epidermidis* is a major cause of device-associated infections [51]. Sub-MIC antibiotics induced biofilm formation in *S. epidermidis* primarily through poly-N-acetylglucosamine (PNAG) synthesis and eDNA-mediated mechanisms [18,41]. Rachid et al. [18] demonstrated that sub-MIC tetracycline and quinupristin-dalfopristin strongly upregulated the icaADBC operon, leading to enhanced PNAG production and biofilm formation. Kaplan et al. [41] reported strain-dependent biofilm induction by sub-MIC vancomycin and tigecycline mediated by either PNAG or eDNA depending on baseline biofilm capacity. Wang et al. [42] showed that macrolides including erythromycin, azithromycin, and clarithromycin induced biofilm formation independently of *ica* gene operon through increased expression of the autolysin *atlE* gene and enhanced eDNA release. These findings suggest that sub-MIC antibiotics stimulate biofilm formation in *S. epidermidis* through both PNAG-dependent and PNAG-independent mechanisms, although the upstream signaling pathways and regulatory networks linking antibiotic stress to these responses remain largely unresolved.

### 3.12. Staphylococcus aureus

*S. aureus*, especially methicillin-resistant *S. aureus* (MRSA), strains are problematic in hospital settings and a frequent cause of medical device infection and skin and soft tissue infections [52]. In *S. aureus*, sub-MIC β-lactams and protein synthesis inhibitors enhance biofilm formation primarily through autolysis-dependent eDNA release and surface protein regulation. Kaplan et al. [15] showed that methicillin-induced biofilms were DNase- and proteinase-sensitive but independent of PNAG. Mlynek et al. [14] demonstrated that amoxicillin-induced eDNA release was necessary but not sufficient for biofilm formation in epidemic MRSA USA300 lineage strains. Transcriptomic analyses further implicated multiple TCS and stress-response pathways in antibiotic-induced biofilm regulation [43]. In addition to autolysis-dependent eDNA release, ciprofloxacin 0.25× MIC increased the expression of fibronectin-binding proteins (FnBPs) in highly quinolone-resistant strains, enhancing surface adhesion. Subsequent studies confirmed a contributory role for FnBPs in MRSA biofilm development [44]. Together, these studies suggest that antibiotic-induced biofilm formation in *S. aureus* arises from a multifactorial interplay between autolysis-mediated eDNA release, adhesin expression, and stress-response pathways, although how these processes are coordinated across different MRSA lineages remains insufficiently characterized.

### 3.13. Enterococcus faecalis

*E. faecalis* is an antimicrobial resistant pathobiont in the gastrointestinal tract with vancomycin-resistant enterococci (VRE) strains being of particular concern [53]. Despite the molecular basis of both biofilm development and drug resistance, the effects of sub-MIC antibiotics on *E. faecalis* biofilm formation have received limited attention. Yu et al. [45] demonstrated that sub-MIC cell wall synthesis inhibitors including ampicillin, oxacillin, ceftriaxone, and fosfomycin significantly enhanced *E. faecalis* biofilm formation through antibiotic-induced lysis and eDNA release whereas antibiotics targeting protein or DNA synthesis had no effect. Moreover, recent evidence indicates that canonical c-di-GMP synthesis in *E. faecalis* remains uncertain, with no known diguanylate cyclases identified and no demonstrated response to extracellular c-di-GMP. Therefore, further studies are required to determine whether c-di-GMP has any direct or indirect role in antibiotic-induced biofilm formation in *E. faecalis* [54]. These findings suggest that antibiotic-induced cell lysis and eDNA release may represent a key mechanism underlying sub-MIC-mediated biofilm enhancement in *E. faecalis*. However, the broader regulatory pathways involved and the role of other antibiotic classes remain largely unexplored.

## 4. Discussion

Beyond bacterial killing, antibiotics are active modulators of bacterial metabolism capable of fundamentally reprogramming cellular behavior. Findings from this systematic review support a model in which sub-MIC antibiotics induce metabolic reprogramming integrated through c-di-GMP-dependent or similar signaling networks shifting bacterial populations from growth-oriented to survival-oriented phenotypes characterized by biofilm formation [29,55]. The molecular basis of this transition is closely linked to the mode of action of antibiotics involving not only classical stress- and damage-response pathways but also genes unrelated to the primary antibiotic targets, with these responses repeatedly intersecting on c-di-GMP-regulated pathways across diverse organisms [19]. In *P. aeruginosa*, for example, aminoglycoside-induced biofilm formation involved coordinated regulation of phosphodiesterase activity, small regulatory RNAs, and quorum sensing systems [19,28,33]. In *C. difficile*, biofilm development is regulated through c-di-GMP-regulated surface proteins, which enhanced eDNA accumulation [56]. Even in species lacking canonical c-di-GMP signaling, such as staphylococci, sub-MIC antibiotics activated alternative c-di-GMP-like regulatory circuits that produced increased biofilm formation [57]. Together, these observations reinforce the concept that c-di-GMP operates as a conserved functional hub within a multilayered regulatory network integrating diverse stress signals into coordinated biofilm response. Sub-MIC antibiotic exposure is common including clinical under-dosing, pharmacokinetic gradients within infected tissues, medical device-associated infections, wastewater contamination, and agricultural antibiotic use. Furthermore, low-dose cephaloridine induces the cps operon responsible for colanic acid biosynthesis in the murine gut, highlighting the physiological relevance of sub-MIC antibiotic responses in host environments [58]. These exposures can promote biofilm formation and persistence across One Health clinical, environmental, and animal reservoirs, thereby contributing to chronic infection and evolution of antimicrobial resistance.

The regulation of biofilm formation through c-di-GMP signaling is highly complex due to the presence of multiple diguanylate cyclases (DGCs) and phosphodiesterases (PDEs) within many bacterial genomes [26]. Numerous bacteria encode GGDEF-, EAL-, and HD-GYP-domain proteins, suggesting that biofilm regulation depends not only on global intracellular c-di-GMP levels but also on spatiotemporally localized c-di-GMP pools [26,59]. In *P. aeruginosa*, multiple PDEs contribute to antibiotic-induced biofilm formation, indicating a complex regulatory network linking antibiotic stress to c-di-GMP turnover [19,28,32]. The persistence of biofilm induction in *arr* mutants further highlights functional redundancy among c-di-GMP regulators [28,32]. Moreover, opposing regulation of *arr* and *PA2200* following sub-MIC tobramycin exposure suggests that distinct enzymes modulate different c-di-GMP pools [60]. Comparative genomic analyses further support this concept, as the *P. aeruginosa* PA14 genome encodes an extensive repertoire of predicted diguanylate cyclases and phosphodiesterases, including 18 GGDEF-, 5 EAL-, 16 GGDEF/EAL-, and 3 HD-GYP-domain proteins, thereby providing multiple parallel pathways capable of modulating c-di-GMP levels during antibiotic stress [61,62]. Beyond antibiotic responses, specific DGCs and PDEs regulate discrete stages of biofilm development, including attachment, maturation, and dispersal, while hybrid GGDEF/EAL proteins such as MucR provide additional regulatory flexibility by switching between DGC and PDE activity depending on cellular context [63,64,65,66,67,68]. Collectively, these findings highlight c-di-GMP signaling as a highly modular, spatially organized, and context-dependent regulatory network rather than a simple linear pathway.

Importantly, c-di-GMP regulates biofilm matrix organization and stability at transcriptional, post-transcriptional, and post-translational levels by controlling the production of key biofilm matrix components, including EPS, pili, fimbriae, protein secretion systems, extracellular proteins, and eDNA [26,69,70,71,72]. c-di-GMP directly regulates EPS biosynthesis, although the underlying mechanisms differ among species [73,74,75]. For example, c-di-GMP promotes PNAG production in *E. coli* by stabilizing the interaction between the inner membrane proteins PgaC and PgaD [76], whereas PNAG synthesis in staphylococci is mediated by the icaADBC operon independently of c-di-GMP [77]. In *C. difficile*, elevated c-di-GMP suppresses the metalloprotease PPEP-1 through riboswitch-mediated regulation, stabilizing surface adhesins and promoting biofilm formation [40,78]. Although penicillin-binding proteins (PBPs) are not direct c-di-GMP targets, cefotaxime-responsive PBPs, particularly PBP1b, have been implicated in antibiotic-induced biofilm formation in *Salmonella enterica*, *E. coli*, and *Listeria monocytogenes*, likely through indirect effects on cell wall homeostasis [36,79,80]. Likewise, in *S. aureus*, sub-MIC fluoroquinolones enhance fibronectin-binding protein (FnBP)-mediated adhesion and biofilm formation [44,81]. This FnBPA-dependent biofilm formation requires the SaeRS two-component system which is regulated by the global metabolic regulator CodY [82,83]. Notably, CodY intersects with c-di-GMP-associated signaling pathways in several low G + C Gram-positive bacteria, including *C. difficile* and *L. monocytogenes* [84,85].

c-di-GMP signaling is further integrated with QS and TCS, linking intracellular signaling with environmental sensing and population-level behavior. In *Vibrio cholerae*, QS activation of HapR lowers intracellular c-di-GMP levels and represses biofilm formation [86], while c-di-GMP-responsive transcriptional regulators such as vpsT activate biofilm-associated genes [87]. In *Burkholderia cenocepacia*, the QS signal BDSF directly binds the hybrid GGDEF/EAL protein RpfR, stimulating phosphodiesterase activity and lowering c-di-GMP to regulate biofilm formation and motility [88]. In *V. cholerae*, the response regulator VpsR directly binds c-di-GMP to activate biofilm-associated genes, while the VxrA/VxrB TCS is also influenced by intracellular c-di-GMP levels [89,90]. In *P. aeruginosa*, elevated c-di-GMP levels inhibit the histidine kinase RetS, thereby activating the GacS/GacA signaling cascade and promoting upregulation of the small RNAs RsmY/Z. These small RNAs repress the translational regulator RsmA, leading to enhanced EPS production, reduced motility and increased sessility [91]. Surface-sensing systems such as Wsp further amplify c-di-GMP synthesis through activation of diguanylate cyclase WspR, thereby reinforcing biofilm development following surface attachment [92].

Overall, the studies examined and discussed in this review support a model in which sub-MIC antibiotics function not only as antimicrobial agents, but also as physiological and metabolic stressors that reshape bacterial behavior through interconnected signaling networks. In this framework, antibiotic-induced metabolic perturbation, nutrient limitation, stress adaptation, and persistence converge on c-di-GMP-dependent regulatory systems that coordinate the transition from planktonic growth to biofilm-associated survival states. Rather than acting as a simple linear signaling pathway, c-di-GMP operates as a highly modular integrative hub linking environmental sensing, cellular physiology, and multicellular biofilm development. This systems-level perspective provides a mechanistic framework for understanding how sub-MIC antibiotic exposure promotes biofilm formation and persistence across diverse bacterial taxa.

Despite these advances, several important questions remain unresolved. The precise mechanisms linking specific antibiotic classes to defined c-di-GMP regulatory modules are still poorly understood, and the relative contributions of metabolic state, stress signaling, and direct regulatory interactions in controlling c-di-GMP dynamics remain unclear. In addition, most current studies rely on bulk measurements of c-di-GMP, which may obscure important spatial and temporal heterogeneity within bacterial populations and mature biofilms. Future studies integrating metabolomics, transcriptomics, single-cell c-di-GMP imaging, and targeted genetic perturbation approaches will therefore be essential for dissecting these complex regulatory interactions and identifying conserved mechanistic principles underlying antibiotic-induced biofilm formation. From a translational perspective, these findings raise important concerns regarding antibiotic dosing strategies and suggest that conventional antimicrobial therapies may unintentionally promote biofilm-associated survival at sub-inhibitory concentrations. Targeting c-di-GMP metabolism, localized signaling nodes, or key downstream effectors may therefore provide promising strategies to disrupt biofilms while minimizing selective pressure for resistance. Future anti-biofilm approaches may benefit from combining conventional antibiotics with therapies that interfere with c-di-GMP signaling, stress-adaptive pathways, or persister-associated metabolic states.

## 5. Conclusions

Bacterial exposure to sub-inhibitory concentrations of antibiotics is a biologically active signal that fundamentally reprograms bacterial physiology and regulatory networks toward biofilm-associated lifestyles. Across diverse bacterial pathogens, sub-MIC antibiotics consistently promoted biofilm development through convergent adaptive strategies that enhanced matrix production, reduced motility, increased surface adhesion, and facilitated stress-adaptive physiological states. Although the precise molecular mechanisms varied across bacterial species and antibiotic classes, the pathways involved repeatedly intersected with c-di-GMP-mediated or c-di-GMP-associated regulatory systems, positioning c-di-GMP as a central integrative signaling hub linking antibiotic-induced stress to biofilm development and persistence.

## Figures and Tables

**Table 1:** c-di-GMP involvement, signaling components, and regulatory mechanisms in sub-MIC antibiotic-induced biofilm formation in Gram-negative bacteria.

Bacterial Species [Ref]	Antibiotic (s)	c-di-GMP Involvement ^1^	DGCs Reported	PDEs Reported	Primary Regulatory Mechanism ^2^	Key Regulatory Pathways	Key Biofilm Matrix Components
*Pseudomonas aeruginosa* [9,19,28,32,33,34]	Aminoglycosides, β-lactams	Direct	-	arr PA2200	Post-translational	OprF-SigX pathways, QS networks, PrrF sRNA	Alginate, Pel/Psl EPS, eDNA
*Pseudomonas putida* [10]	Tetracycline	Direct	WspR	-	Transcriptional and post-translational	RpoS-Wsp signaling	EPS
*Escherichia coli* [11,21]	Erythromycin, tetracycline, chloramphenicol, streptomycin, β-lactams	Direct	YdeH	-	Post-transcriptional	ppGpp-c-di-GMP crosstalk	PNAG, colanic acid
*Klebsiella pneumoniae* [22,35]	Fluoroquinolone, tetracyclines	Direct	yfiN	mrkJ	Post-translational	c-di-GMP-MrkH signaling	EPS, type III fimbriae, eDNA
*Acinetobacter baumannii* [37]	Cephalosporins	Indirect	-	-	-	Matrix remodeling	eDNA, proteins, lipids, OMVs
*Haemophilus influenzae* (NTHi) [8,46]	β-lactams	Indirect	-	-	-	Autolysis-driven	eDNA, glycogen
*Salmonella enterica* [13,36]	Cephalosporins	Indirect	-	-	-	PBP1b-mediated signaling	EPS

Abbreviations: DGCs, diguanylate cyclases; PDEs, phosphodiesterases; EPS, extracellular polymeric substances; ppGpp, guanosine pentaphosphate; QS, quorum sensing; PNAG, poly-N-acetylglucosamine; OMVs, outer membrane vesicles; PBP, penicillin-binding protein; PrrF, iron-regulated small regulatory RNA. ^1^ Direct involvement indicates studies reporting altered intracellular c-di-GMP levels or genetic/functional characterization of diguanylate cyclases (DGCs), phosphodiesterases (PDEs), riboswitches, or c-di-GMP-binding effectors. Indirect involvement indicates inferred c-di-GMP participation based on biofilm phenotypes or involvement of pathways known to intersect with c-di-GMP signaling. ^2^ Primary regulatory mechanism reflects the dominant mechanism reported in the referenced studies; multiple regulatory layers may coexist.

**Table 2:** c-di-GMP involvement, signaling components, and regulatory mechanisms in sub-MIC antibiotic-induced biofilm formation in Gram-positive bacteria.

Bacterial Species [Ref]	Antibiotic (s)	c-di-GMP Involvement ^1^	DGCs Reported	PDEs Reported	Primary Regulatory Mechanism ^2^	Key Regulatory Pathways	Key Biofilm Matrix Components
*Clostridioides difficile* [38,39,40,47]	Glycopeptide, nitroimidazoles	Direct	GGDEF-domain proteins	EAL-domain proteins	Post-transcriptional and post-translational	c-di-GMP riboswitches, LuxS-QS system	eDNA, cell surface proteins
*Enterococcus faecalis* [45]	β-lactams, fosfomycin	Absent	-	-	Post-translational	Antibiotic-induced lysis	eDNA
*Staphylococcus epidermidis* [18,41,42]	Tetracyclines, macrolides, glycopeptides	Absent	-	-	Transcriptional/post-translational	icaADBC operon, autolysis-mediated eDNA release	PNAG, eDNA
*Staphylococcus aureus* [14,15,43,44]	β-lactams, tetracyclines, fluoroquinolones	Absent	-	-	Post-translational	Autolysis, TCS	eDNA, FnBPs

Abbreviations: DGCs, diguanylate cyclases; PDEs, phosphodiesterases; EPS, extracellular polymeric substances; PNAG, poly-N-acetylglucosamine; QS, quorum sensing; AI-2, autoinducer-2; TCS, two-component systems. ^1^ Direct involvement indicates studies reporting altered intracellular c-di-GMP levels or genetic/functional characterization of diguanylate cyclases (DGCs), phosphodiesterases (PDEs), riboswitches, or c-di-GMP-binding effectors. Indirect involvement indicates inferred c-di-GMP participation based on biofilm phenotypes or involvement of pathways known to intersect with c-di-GMP signaling. Absent indicates that canonical c-di-GMP signaling is not present as in staphylococci which lack functional GGDEF/EAL/HD-GYP-domain-containing proteins. ^2^ Primary regulatory mechanism reflects the dominant mechanism reported in the referenced studies; multiple regulatory layers may coexist.

## Data Availability

Data sharing is not applicable to this article as no datasets were generated or analyzed during the current study.
